# Effects of acute hypoventilation and hyperventilation on exhaled carbon monoxide measurement in healthy volunteers

**DOI:** 10.1186/1471-2466-9-51

**Published:** 2009-12-23

**Authors:** Franco Cavaliere, Carmen Volpe, Riccardo Gargaruti, Andrea Poscia, Michele Di Donato, Giovanni Grieco, Umberto Moscato

**Affiliations:** 1 Institute of Anaesthesia and Intensive Care, Catholic University of the Sacred Heart, Rome, Italy; 2 Institute of Hygiene, Catholic University of the Sacred Heart, Rome, Italy

## Abstract

**Background:**

High levels of exhaled carbon monoxide (eCO) are a marker of airway or lung inflammation. We investigated whether hypo- or hyperventilation can affect measured values.

**Methods:**

Ten healthy volunteers were trained to achieve sustained end-tidal CO2 (etCO2) concentrations of 30 (hyperventilation), 40 (normoventilation), and 50 mmHg (hypoventilation). As soon as target etCO2 values were achieved for 120 sec, exhaled breath was analyzed for eCO with a photoacoustic spectrometer. At etCO2 values of 30 and 40 mmHg exhaled breath was sampled both after a deep inspiration and after a normal one. All measurements were performed in two different environmental conditions: A) ambient CO concentration = 0.8 ppm and B) ambient CO concentration = 1.7 ppm.

**Results:**

During normoventilation, eCO mean (standard deviation) was 11.5 (0.8) ppm; it decreased to 10.3 (0.8) ppm during hyperventilation (p < 0.01) and increased to 11.9 (0.8) ppm during hypoventilation (p < 0.01). eCO changes were less pronounced than the correspondent etCO_2 _changes (hyperventilation: 10% Vs 25% decrease; hypoventilation 3% Vs 25% increase). Taking a deep inspiration before breath sampling was associated with lower eCO values (p < 0.01), while environmental CO levels did not affect eCO measurement.

**Conclusions:**

eCO measurements should not be performed during marked acute hyperventilation, like that induced in this study, but the influence of less pronounced hyperventilation or of hypoventilation is probably negligible in clinical practice

## Background

In the human body, carbon monoxide (CO) has two origins: the exogenous source is the absorption from tobacco smoke and polluted air, the endogenous one is the breakdown of heme molecules by heme oxidase (HO) [1,2]. Exogenous CO increases in relation with the exposure to tobacco smoke and polluted air. Endogenous CO grows in the case of increased heme catabolism, caused by hemolysis, or by the synthesis of the isoenzyme HO-1, also known as stress-inducible heat shock protein 32, which is upregulated by a variety of stressors, such as cytokines, hypoxia, and reactive oxygen species. In all cases, the increased CO in the body results in increased CO levels in exhaled breath (eCO). Consequently eCO measurements may be useful to evaluate smoking abstinence [3], exposition to polluted air [4], oxidative stress caused by anaesthesia or surgery [5], severe sepsis [6], stable asthma and asthma exacerbations [7,8], chronic obstructive pulmonary disease (COPD) [9], hemolysis in children with sickle cell disease [10], and upper [11] and lower airway inflammation [12]. Table 1 reports some examples of eCO levels in smoking healthy subjects and patients with significant issues.

**Table 1:** Exhaled CO levels in smoking healthy subjects and patients with significant issues.

Authors	Diseases or smoking	Exhaled CO level (ppm)		N. Subjects
		
		Mean	**Range or S.D. or 95% C.I**.	
**Deveci et al (2004) **[27]	Smoking	17.13	± 8.5	243
**Carpagnano et al (2003)**[28]	Smoking	16.70	± 5.5	31
**Montuschi et al. (2001) **[9]	COPD	7.40	± 1.9	15
**Montuschi et al. (2001) **[9]	COPD + Smoking	20.0	± 2.6	15
**Biernacki et al (2001) **[12]	LRTI	5.20	± 0.5	35
**Yamaya et al (1998) **[29]	URTI	5.60	± 0.4	20
**Ohara Y et al (2006) **[7]	Asthma	5.10	± 0.4	22
**Kiyoshi et al (1997) **[30]	Asthma	5.60	± 0.6	12
**Paredi et al (1999) **[31]	CF	6.70	± 0.6	29
**Horvath et al. (2003) **[32]	Bronchiectasis	5.00	3.0 - 15.0	31
**De Las Heras et al (2003) **[33]	SBP	7.00	± 0.5	9

COPD chronic obstructive pulmonary disease; LRTI lower respiratory tract infection; URTI upper respiratory tract infections; CF cystic fibrosis; SBP spontaneous bacterial peritonitis;

Independently from the amount of CO produced by a patient, his/her eCO levels may be influenced by the condition in which measurements are performed. For instance eCO can temporarily increase during oxygen therapy because hyperoxia facilitates CO release from carboxyhemoglobin [13-15]. Acute hyperventilation may decrease eCO values by dilution; in addition theoretical models suggest that alveolar ventilation may affect CO stores by varying the partial pressure of this gas in the alveoli and consequently in blood and tissues. In that case a proportionate amount of CO should be released or stored in order to achieve a new equilibrium [16]. This process can take several hours, meanwhile eCO measures may be poorly representative of CO production.

Changes of ventilatory pattern can potentially occur near eCO measurements. Patients can be induced to hyperventilate by the procedure of breath sampling itself [17]; furthermore, acute ventilatory changes are not infrequent in asthma exacerbations, sepsis, or during assisted mechanical ventilation. The aim of this study was to evaluate whether acute hypo- or hyperventilation can affect eCO measurements. Secondarily, we evaluated the influence of environmental CO and breath sampling procedure on eCO measurements.

## Methods

The study was carried out in the Catholic University of the Sacred Heart in Rome. Measurements were performed in duplicate in two laboratories situated in different buildings, the first one in the Surgical Intensive Care Unit (A), the second in the institute of Hygiene (B). After the approval of local Ethic Committee and informed consent, 10 healthy volunteers (5 men and 5 women) were enrolled in the study. Table 2 reports theirs anthropometric and lung functions values.

**Table 2:** Anthropometric and lung function values as means (standard deviations) of the healthy volunteers enrolled in this study.

	Men	Women
**AGE (anni)**	31.40 (12.30)	28.60 (12.20)
**BMI (Kg/m^2^)**	24.19 (1.41)	20.88 (3.10)
**FEV_1_(L)**	3.76 (0.40)	2,62 (0.64)
**FVC (L)**	4.23 (0.48)	3.02 (0.67)
**FEV_1_/FVC (%)**	88.95 (1.85)	86.57 (2.10)
**PFER (L/s)**	8.57(0.96)	5.32 (1.33)

BMI body mass index; FEV_1 _forced expiratory volume in one second; FVC forced vital capacity; FEV_1_/FVC forced expiratory volume in one second/forced vital capacity; PEFR peak expiratory flow rate;

Exclusion criteria were: a) active or passive tobacco smoke, b) chronic respiratory diseases, and c) acute respiratory diseases occurred in the past four months.

In each subject, eCO was measured at end tidal CO_2 _(etCO_2_) values of 30, 40, and 50 mmHg in A and B settings. At etCO_2 _values of 30 and 40 mmHg, exhaled gases were collected both after normal inspiration and after maximum inspiration. Measurements at 50 mmHg were only performed after normal inspiration in order to avoid a brisk etCO_2 _decrease. Measurement were performed twice in each setting.

**Figure 1:**
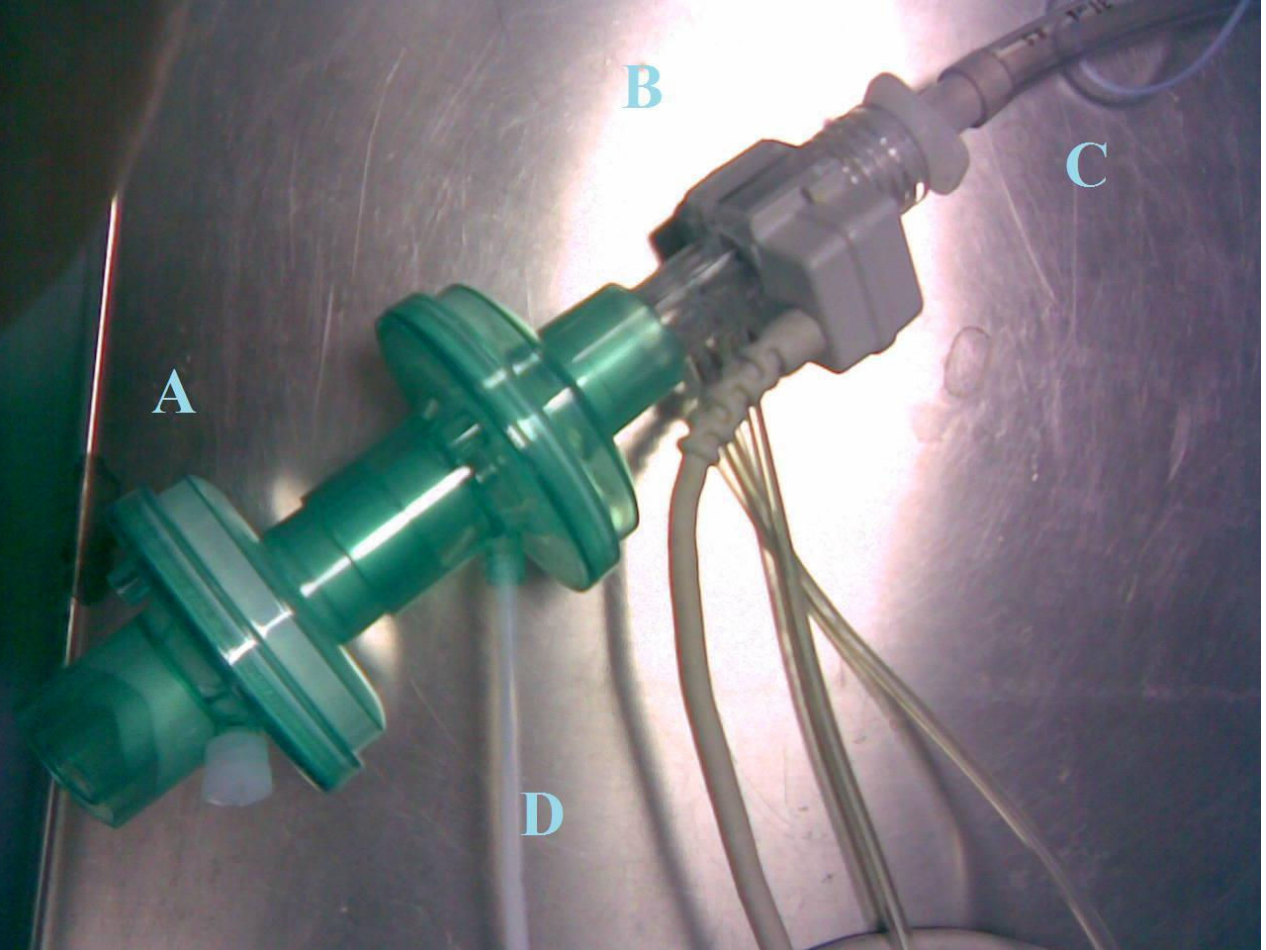
**The device assembled for breath sampling and etCO_2 _measurement**. The HME filter (A); the cell of the main stream capnometer CosmoPlus mod. 8100 (Novametrix Medical Systems Inc. Connecticut, USA) in order to measure etCO_2 _(B); a Mallinkrodt tracheal tube with an internal diameter of 9 mm in order to prevent any risk of collecting external air during sampling for eCO measurements (C). The tube 1 m long and with an internal diameter of 3 mm through which breath samples were suctioned (D).

Subjects were asked to breath through a mouth piece in a device (figure 1) including a) a HME bacterial/viral filter (DAR Barrierbac S, Mallinkrodt DAR, Italy); b) the cell of the main stream capnometer CosmoPlus mod. 8100 (Novametrix Medical Systems Inc. Connecticut, USA) in order to measure etCO_2_; c) a tracheal tube (Mallinkrodt Inc, UK) with an internal diameter of 9 mm in order to prevent any risk of collecting external air during sampling for eCO measurements. The HME filter (internal volume 35 mL) protected the capnometer cell from condensation and allowed gas sampling for eCO measurements from the capnometry port. Initially, subjects were trained: a) to perform a prolonged expiration that lasted not less than 16 seconds, and b) to hypoventilate or hyperventilate in order to maintain a target etCO_2 _value on the display of the capnometer. Successively, they were asked to achieve the etCO_2 _values of 30, 40, and 50 mmHg in random sequence. As soon as the target etCO_2 _value was maintained within a range of ± 2 mmHg for 120 sec, the patients were asked to perform a prolonged expiration and exhaled gases were sampled for eCO analysis.

Breath samples were suctioned through a tube 1 m long and with an internal diameter of 3 mm by the photoacoustic spectrometer utilized for CO analysis; 80 mL were collected in 10 sec. Sampling was started by an operator three seconds after the beginning of a prolonged expiration lasting 16 seconds or more. It was initially assessed with capnography that an interval of two seconds was long enough to avoid anatomical dead space sampling. As a further precaution, the operator looked at the capnogram displayed by Cosmo plus during gas sampling; if sampling started before phase C (alveolar plateau), the procedure was repeated. The etCO_2 _value measured before (pre-test etCO_2_) and during (test etCO_2_) each eCO measurements and the peripheral arterial oxygen saturation (SpO_2_) were registered. Before each session of the study, the capnometer was calibrated and environmental CO concentration was measured.

eCO was measured with a photo-acoustic spectrometer field gas monitor Innova 1312 (LumaSense Technologies A/S, Denmark) equipped with a filter for CO analysis and with the software 7300 (LumaSense Technologies A/S, Denmark). The instrument is based on the photo-acoustic effect in the infra-red wave-lenghts[18]. Sampled CO absorbs light energy at a specific wave length, emitted by spectrometer, and transforms it into kinetic energy; this process causes local heating and pressure waves resulting in sounds that can be detected by a microphone. The photo-acoustic field gas monitor suctions gaseous samples by a pump to fill the photo-acoustic cell in which measurements are performed. Dead space, water-vapor's (or other gases) and temperature interference were automatically compensated. Detection limit was ≥ 0.02 ppm (part per million), with Sample Integration Times (S.I.T.) fixed at 5 seconds and multiplication factor 1.

The statistical analysis was carried out with the software "Statistica for Windows" (StatSoft Inc, USA) and STATA Intercooled v. 9.2 software for MacIntosh (Stata Co.; College Station Lakewag, TX, USA). Values are reported as means (standard deviations). Major deviations from normal distribution were ruled out by graphic inspection and by Shapiro-Wilks's test. The Kruskal-Wallis (K-W) equality-of-populations rank test (adjusted with Bonferroni at level of significance p ≤ 0.05) was employed to assess real difference of variation between lab A vs lab B.

Statistical analysis on eCO values was performed with ANOVA for repeated measures; planned comparisons were tested with linear contrasts. Factors taken into account were setting (lab A Vs B) and etCO_2 _(30 Vs 40 Vs 50 mmHg); planned comparisons were hypoventilation Vs normoventilation, hyperventilation Vs normoventilation, normal inspirations Vs deep inspiration. ANOVA for repeated measures was also applied to SpO2 values. The factor taken into account was etCO_2_; planned comparisons were hypoventilation Vs normoventilation and hyperventilation Vs normoventilation. Finally, pre-test and test etCO_2 _values were compared with paired t-test.

## Results

eCO analysis showed a good level of reproducibility. The mean difference between duplicate measurements was 0.11 (0.20) ppm. Environmental CO concentration was 0.8 ppm in lab A and 1.7 ppm in lab B.

eCO values are reported in table 3. In comparison with normoventilation, eCO values were lower during hyperventilation and higher during hypoventilation (p < 0.01 in both cases); difference was more pronounced between normo- and hyperventilation (mean value: 1.2 ppm) than between normo- and hypoventilation (mean value: 0.4 ppm). No significant difference was observed between measurements performed in lab A and B (K-W test), while measured eCO levels were significantly lower when gas sampling was performed after a deep inspiration than after a normal inspiration (p < 0.01).

**Table 3:** Exhaled carbon monoxide values registered in laboratory A and B.

Et-CO_2 _target	30 mmHg (hyperventilation)		40 mmHg (normoventilation)		50 mmHg (hypoventilation)
**Inspiration**	**Normal**	**Deep**	**Normal**	**Deep**	**Normal**

**Lab A (0.8 ppm)**	10.2(0.9)	9.6(0.8)	11.4(0.8)	10.7(0.8)	11.8(0.9)
**Lab B (1.7 ppm)**	10.3(0.7)	9.7(0.7)	11.6(0.8)	10.8(0.8)	11.9(0.7)
**Total**	10.3(0.8)	9.7(0.8)	11.5(0.8)	10.8(0.8)	11.9(0.8)

Means (standard  deviations) of exhaled carbon monoxide (eCO) values registered in laboratory A and B (environmental eCO level 0.8 and 1.7 ppm, respectively). Values are reported as particles per milion (ppm)  .

Statistical analysis: ANOVA: LabA Vs Lab B non significant; hyperventilation Vs normoventilation p < 0.01; hyperventilation Vs normoventilation p < 0.01; normal inspiration Vs deep inspiration p < 0.01.

Table 4 reports SpO_2 _and etCO_2 _values. In comparison with normoventilation, SpO_2 _increased during hyperventilation and decreased during hypoventilation (p < 0.01 in both cases). Test etCO_2 _registered during the prolonged expiration for gas sampling were significantly higher than pre-test etCO_2 _(p < 0.01).

**Table 4:** Exhaled carbon monoxide, end tidal CO2, and peripheral oxygen saturation.

Et-CO2 target	30 mmHg (hyperventilation)		40 mmHg (normoventilation)		50 mmHg (hypoventilation)
**Inspiration**	**Normal**	**Deep**	**Normal**	**Deep**	**Normal**

**eCO (ppm)**	10.3 (0.8)	9.7 (0.8)	11.5 (0.8)	10.8 (0.8)	11.9 (0.8)
**Pre-test etCO_2 _(mmHg)**	29.4 (1.6)	28.7 (1.6)	39.4 (1.7)	39.2 (1.7)	49.1 (1.7)
**Test etCO_2 _(mmHg)**	37.8 (2.5)	35.6 (2.8)	46.1 (3.3)	43.0 (2.9)	51.6 (3)

**SpO_2 _(%)**	99 (1)		98 (1)		96 (2)

Means (standard deviations) of exhaled carbon monoxide (eCO) values, end tidal CO_2 _values measured before and during breath sampling (respectively pre-test etCO_2 _and test etCO_2_), and peripheral oxygen saturation (SpO_2_).

Statistical analysis. EtCO_2_: pre-test Vs test p < 0.01. SpO_2_: ANOVA p < 0.01; hyperventilation Vs normoventilation p < 0.01; hyperventilation Vs normoventilation p < 0.01.

## Discussion

In general ventilation patterns can influence quantification of volatile analytes in exhaled breath and should be controlled to ensure representative analyses [17]. In this study we evaluated whether acute hypo- or hyperventilation can significantly influence eCO measurements. This hypothesis was only partially confirmed because hyperventilation decreased eCO, but to a much lesser extent than etCO_2_; in practice, eCO decreased by 10% while etCO_2 _decreased by 25%. Hypoventilation had an even lesser effect, causing a small and clinically negligible eCO increase (3%) while etCO_2 _increased by 25%. We also found that eCO measurements were not affected by environmental CO, but decreased slightly if a deep inspiration is taken before breath sampling.

Changes of ventilatory pattern are common in patients affected by respiratory diseases, particularly during mechanical ventilation; in addition, the procedure of breath sampling itself may induce patients to hyperventilate [17]. Acute hypo- and hyperventilation cause proportional changes of the levels of many analytes, such as CO_2_, isoprene, and ethane, in exhaled breath [17-19]. By contrast our results point out that hypo- and hyperventilation have small impact on eCO measurements; this is in agreement with the observation reported by other Authors that ventilatory changes affects eCO and etCO_2 _differently [17].

Our results are apparently in contrast with theoretical predictions. In steady state, eCO is strictly correlated with the endogenous production of CO and with carboxyhaemoglobin blood levels [20], but these relationships are influenced by alveolar ventilation, as well as by the environmental CO levels, by the diffusing capacity of the lung for CO, and by the oxygen partial pressure in pulmonary capillaries [16]. By means of a mathematical model, Cobourn and Coll calculated that doubling alveolar ventilation from 4 to 8 liters per minute in a patient with a normal CO production of 0.42 mL per hour would have resulted in a 43% decrease of carboxyhaemoglobin, from 0.37 to 0.21% [16]; a corresponding variation can be hypothesized for eCO. The smaller changes observed in this study are possibly explained because calculations were referred to the new steady state that may be achieved only several hours after ventilatory variations. Cobourn and Coll estimated that the half-life of the process was about 7 hours [16], far longer than the half-life of CO_2 _store variations, which have been estimated around 70 minutes or less with animal models [21,22] and around 35 minutes in men [19]. The longer CO half-life may be explained by the small amount of this gas released every minute through the lungs in comparison with the stores in the human body. Stored CO has been estimated at 10 mL, 8 of which are bound to hemoglobin [23]. The CO released by a healthy man is about 0.4 mL per hours [21] i.e. 7 μL per minute, which corresponds to about 0.07% of body stores and 0.09% of blood content. Conversely, the readily exchangeable CO_2 _stored in the body is about 14 L [24] and the CO_2 _released is about 200-250 mL per minute, which corresponds to about 1.4-1.8% of body stores. As a consequence the stabilization of eCO and blood carboxyhemoglobin levels after ventilatory changes requires many hours. Hypothetically, if in this study hypoventilation or hyperventilation had been prolonged for a time interval long enough to approximate the steady state, eCO changes would have possibly shown the same magnitude of etCO_2_changes.

Our results stress the importance of standardizing the procedure for measuring eCO. We observed that taking a deep or normal inspiration can affect eCO levels, other Authors reported that measuring after a breath hold may result in higher eCO values than sampling without breath hold [25] In addition, our data suggest that, even in healthy subjects, a deep expiration may recruit pulmonary alveoli that are poorly represented in a normal expiration since a significant difference was observed between etCO_2 _values measured immediately before (pre-test) and during (test) breath sampling. Of note, pulmonary heterogeneity strongly affects the relationship between eCO and CO-Hb, [16] which is lost in patients affected by severe pulmonary emphysema or airway obstruction [20,26].

A limit of our study is that we did not evaluate the effects of prolonged ventilatory changes on eCO. In addition, we measured CO values by a photoacoustic spectrometer. To our knowledge this kind of instrument has not been utilized for eCO measurement before. In this study photoacoustic spectrometer performed CO analysis with very high sensitivity and precision on breath samples that were representative of the entire exhaled breath. Theoretically the eCO values obtained with our method may reflect a wider alveolar population in comparison with the end tidal values provided by electrochemical sensors that are utilized for routine eCO measurements. These methodological differences may partly explain why the volunteers enrolled in this study presented relatively high eCO values for non-smokers compared with literature data (Table 5). Other possible explanations were that all the subjects lived in densely populated urban environment and were consequently exposed to high levels of environmental CO and that eCO values are characterized by large interindividual variability [4]; as a matter of fact, eCO levels similar to those observed in this study have been reported in healthy non smokers by other authors [14].

**Table 5:** Exhaled carbon monoxide values in non smoking healthy subjects reported in literature.

Authors	Exhaled CO level (ppm)		N. Subjects
		
	Mean	**Range or S.D. or 95% C.I**.	
**Chatkin J. et al. (2007) **[3]	4.30	± 2.5	152
**Cunnington and Hormbrey (2002) **[34]	1.26	1.14-1.37	366
**Deveci et al (2004) **[27]	3.61	± 2.15	55
**Horvath et al. (2003) **[32]	3.00	0.5-5.0	37
**Laranjeira R. at al (2000) **[35]	2.50	1.0 - 4.0	100
**Montuschi et al. (2001) **[9]	3.00	± 0.3	10
**Scharte et al. (2000) **[36]	1.55	1.2-1.7	6

## Conclusions

While eCO values are scarcely affected by hypoventilation that occurs during measurement, hyperventilation should be avoided because it significantly decreases eCO values. Nonetheless light hyperventilation occurring during measurements may be acceptable because eCO changes are much less pronounced than ventilation changes (reflected by etCO_2_).

## Competing interests

The authors declare that they have no competing interests.

## Authors' contributions

FC and UM conceived of the study and reviewed the manuscript. RG and MDD were involved with data collection and statistical analysis. CV, GG, and AP drafted the manuscript.

All authors read and approved the final manuscript.

## Pre-publication history

The pre-publication history for this paper can be accessed here:

http://www.biomedcentral.com/1471-2466/9/51/prepub
